# Supplementary figures and images for: Metabolomic analysis of bioactive compounds in dill (Anethum graveolens L.) extracts
Source: PeerJ. 2025 Jun 10;13:e19567. doi: 10.7717/peerj.19567 (PMC12164813; doi:10.7717/peerj.19567)

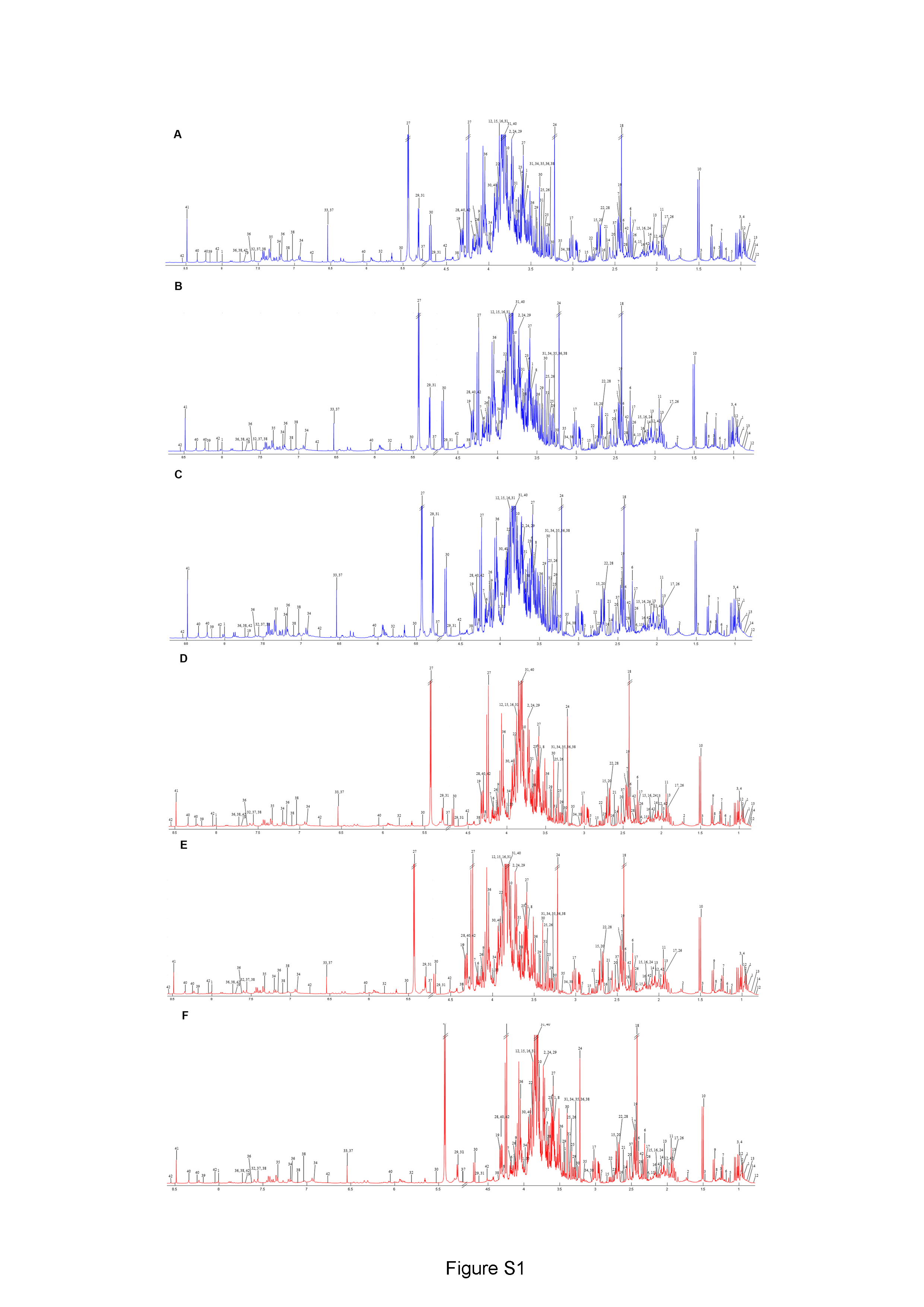

Supplement: Supplemental Information 1 — (A) 27 °C, 2 min; (B) 27 °C, 1 h; (C) 27 °C, 2 h; (D) 90 °C, 2 min; (E) 90 °C, 1 h; and (F) 90 °C, 2 h. Keys: 1, pantothenate; 2, leucine; 3, isoleucine; 4, valine; 5, α-ketoisovalerate; 6, β-hydroxybutyrate; 7, (S)-3-hydroxybutyrate; 8, lactate; 9, threonine; 10, alanine; 11, acetate; 12, α-hydroxyisovalerate; 13, isovalerate; 14, α-ketoisocaproate; 15, homocysteine; 16, methionine; 17, g -aminobutyrate; 18, succinate; 19, pyridoxamine; 20, citrate; 21, aspartate; 22, sarcosine; 23, acetylcholine; 24, 3,7-dimethylurate; 25, proline; 26, sucrose; 27, malate; 28, β-glucose; 29, cellobiose; 30, α-glucose; 31, uracil; 32, fumarate; 33, tyrosine; 34, phenylalanine; 35, tryptophan; 36, pyridoxal; 37, indole-3-lactate; 38, adenine; 39, inosine; and 40, formate; 41, folate. Data are represented in means ± SD (n = 5). [file peerj-13-19567-s001.png]

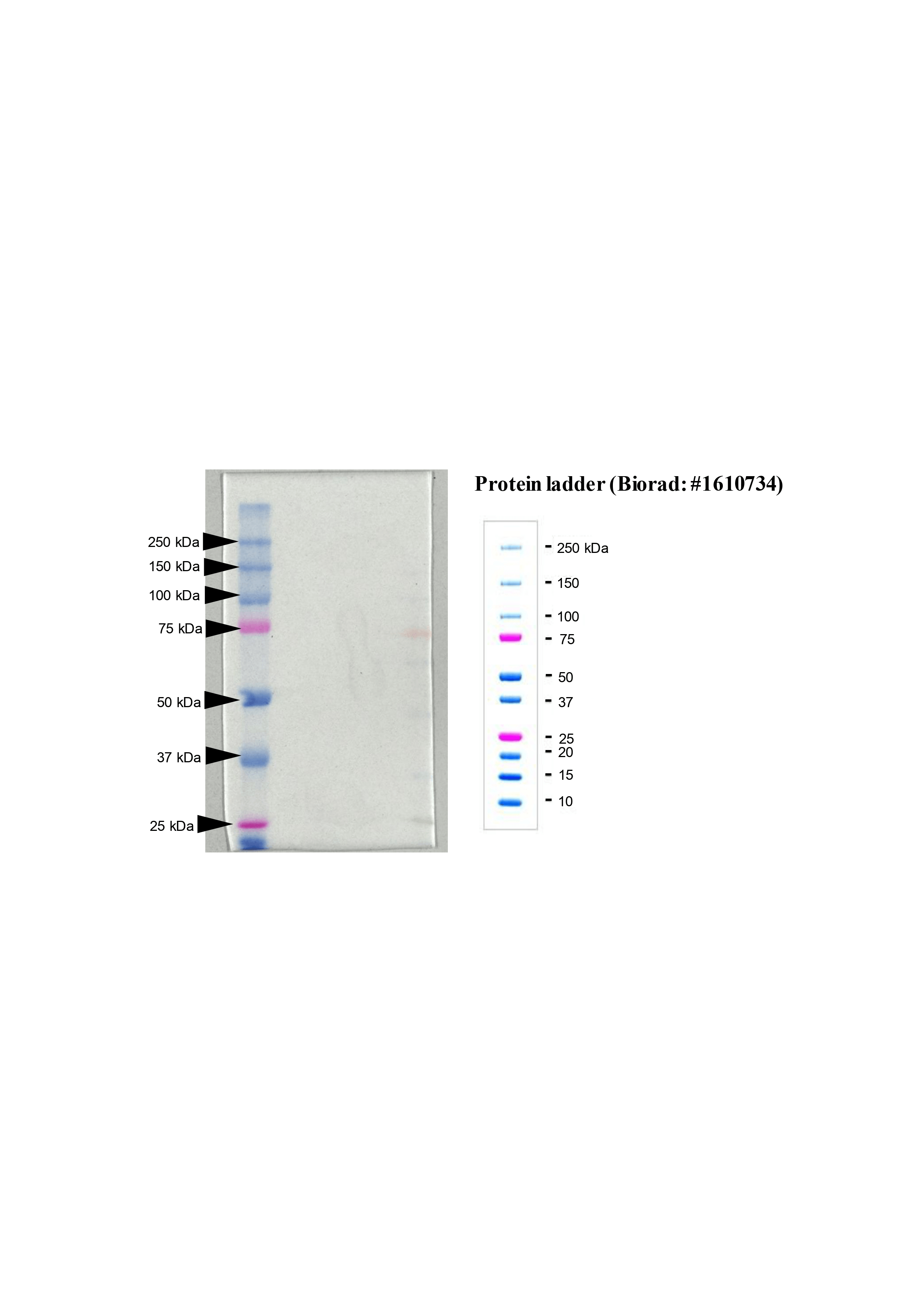

Supplement: Supplemental Information 10 [file peerj-13-19567-s010.zip › Raw data of western blots/WB raw data_1_protein markers.png]

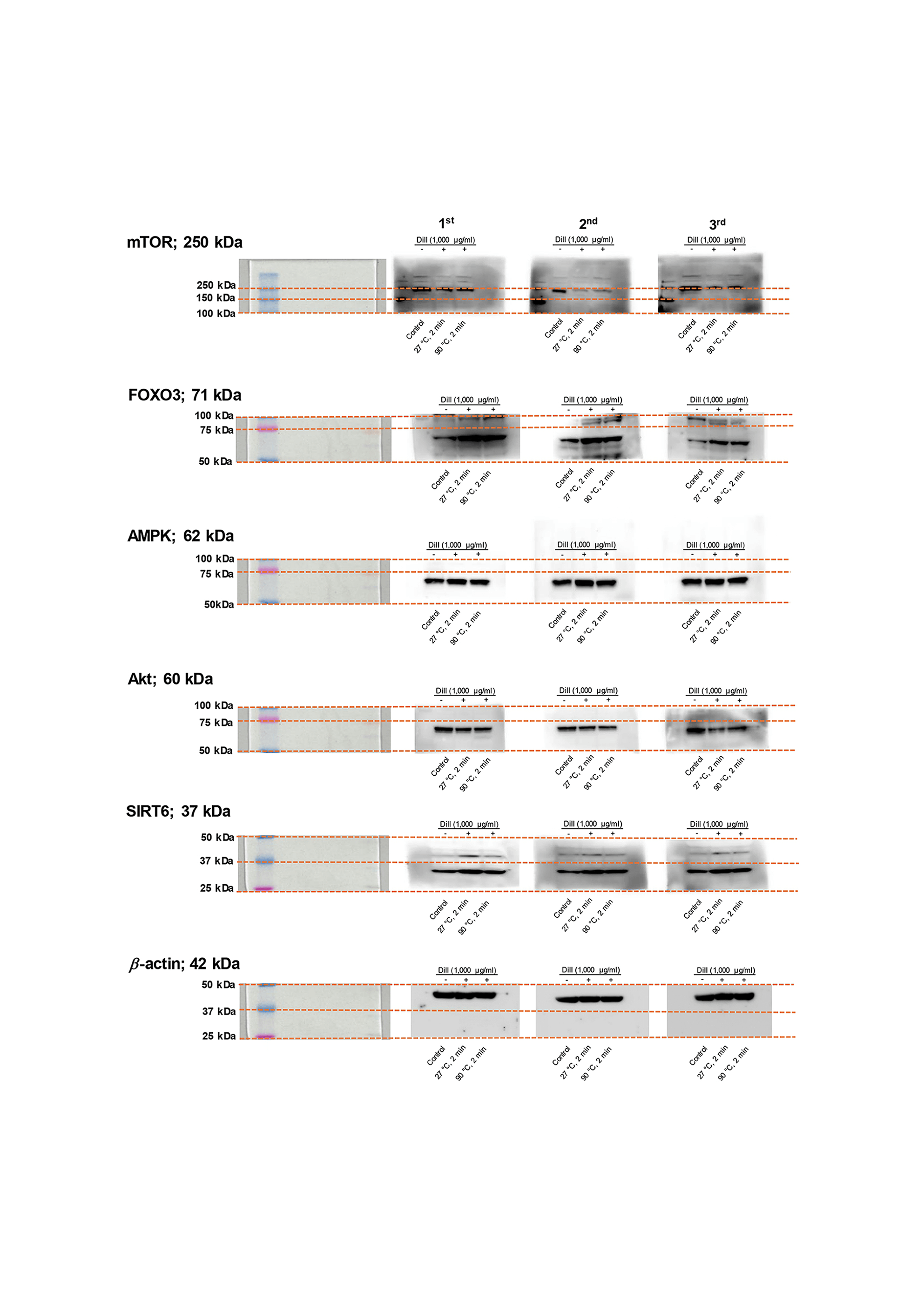

Supplement: Supplemental Information 10 [file peerj-13-19567-s010.zip › Raw data of western blots/WB raw data_2_western blot.png]
