# Supplementary material for: Metabolomic analysis of bioactive compounds in dill (Anethum graveolens L.) extracts
Source: PeerJ. 2025 Jun 10;13:e19567. doi: 10.7717/peerj.19567 (PMC12164813; doi:10.7717/peerj.19567)
Supplement: Supplemental Information 2 [file peerj-13-19567-s002.docx]

**Table S1** Percentage yield of dill leaf extracts

| Extracts | % Extraction yield |
| --- | --- |
| 27 °C, 2 min | 13.52 ± 1.45 |
| 27 °C, 1 h | 14.04 ± 0.93 |
| 27 °C, 2 h | 14.68 ± 0.96 |
| 90 °C, 2 min | 14.42 ± 1.81 |
| 90 °C, 1 h | 14.11 ± 0.69 |
| 90 °C, 2 h | 13.90 ± 0.85 |
