# Supplementary material for: Metabolomic analysis of bioactive compounds in dill (Anethum graveolens L.) extracts
Source: PeerJ. 2025 Jun 10;13:e19567. doi: 10.7717/peerj.19567 (PMC12164813; doi:10.7717/peerj.19567)
Supplement: Supplemental Information 3 [file peerj-13-19567-s003.docx]

**Table S2** 1D and 2D *J*-resolved ^1^H NMR Spectra of metabolic profiles of dill leaf extracts

| **No.** | **ppm** | **Multiplicity**  **by 1D NMR** | **1^st^ tentative metabolites** | **Multiplicity by**  **2D *J*-resolved ^1^H NMR** | **Final tentative metabolites** |
| --- | --- | --- | --- | --- | --- |
| 1 | 0.9291 | s | Pantothenate | s | Pantothenate |
| 2 | 0.9583 | m | Leucine | m | Leucine |
| 3 | 1.001 | d | Isoleucine | d | Isoleucine |
| 4 | 1.04 | d | Valine | d | Valine |
| 5 | 1.1336 | d | *α-*ketoisovalerate | d | *α*-ketoisovalerate |
| 6 | 1.1804 | d | *β*-hydroxybutyrate | d | *β*-hydroxybutyrate |
| 7 | 1.2268 | d | (S)-3-hydroxybutyrate | d | (*S*)-3-hydroxybutyrate |
| 8 | 1.333 | d | Threonine | d | Threonine |
| 9 | 1.3439 | d | Lactate | d | Lactate |
| 10 | 1.4976 | d | Alanine | d | Alanine |
| 11 | 1.627 | s | Unknown1 | s | Unknown1 |
| 12 | 1.9471 | s | Acetate | s | Acetate |
| 13 | 2.001 | m | *α* -hydroxyisovalerate | m | *α*-hydroxyisovalerate |
| 14 | 2.046 | d | Isovalerate | d | Isovalerate |
| 15 | 2.083 | m | *α* -ketoisocaproate | m | *α* -ketoisocaproate |
| 16 | 2.147 | m | Homocysteine | m | Homocysteine |
| 17 | 2.154 | s | Methionine | s | Methionine |
| 18 | 2.199 | s | Unknown2 | s | Unknown2 |

**Table S2** 1D and 2D *J*-resolved ^1^H NMR Spectra of metabolic profiles of dill leaf extracts (continued)

| **No.** | **ppm** | **Multiplicity**  **by 1D NMR** | **1^st^ tentative metabolites** | **Multiplicity by**  **2D *J*-resolved ^1^H NMR** | **Final tentative metabolites** |
| --- | --- | --- | --- | --- | --- |
| 19 | 2.211 | s | Unknown3 | s | Unknown3 |
| 20 | 2.292 | d | *γ*-aminobutyrate | d | *γ*-aminobutyrate |
| 21 | 2.316 | t | Succinate | s | Succinate |
| 22 | 2.4221 | s | Pyridoxamine | s | Pyridoxamine |
| 23 | 2.443 | s | Unknown4 | s | Unknown4 |
| 24 | 2.482 | m | Unknown5 | x | Unknown5 |
| 25 | 2.497 | s | Unknown6 | s | Unknown6 |
| 26 | 2.505 | s | Citrate | s | Citrate |
| 27 | 2.5244 | d | Methylamine | x | Unknown7 |
| 28 | 2.595 | s | Aspartate | s | Aspartate |
| 29 | 2.6698 | dd | Sarcosine | dd | Sarcosine |
| 30 | 2.7441 | s | Acetylcholine | s | Acetylcholine |
| 31 | 3.219 | s | Unknown8 | s | Unknown8 |
| 32 | 3.275 | dd | 3,7-dimethylurate | dd | 3,7-dimethylurate |
| 33 | 3.352 | m | Unknown9 | x | Unknown9 |
| 34 | 3.364 | s | Proline | s | Proline |
| 35 | 3.371 | s | Unknown10 | x | Unknown10 |
| 36 | 3.398 | s | Ethylene glycol dimethyl ether | d | Unknown11 |

**Table S2** 1D and 2D *J*-resolved ^1^H NMR Spectra of metabolic profiles of dill leaf extracts (continued)

| **No.** | **ppm** | **Multiplicity**  **by 1D NMR** | **1^st^ tentative metabolites** | **Multiplicity by**  **2D *J*-resolved ^1^H NMR** | **Final tentative metabolites** |
| --- | --- | --- | --- | --- | --- |
| 37 | 4.24 | d | Sucrose | d | Sucrose |
| 38 | 4.279 | d | Unknown12 | x | Unknown12 |
| 39 | 4.304 | dd | Malate | dd | Malate |
| 40 | 4.422 | m | Unknown13 | x | Unknown13 |
| 41 | 4.487 | d | Unknown14 | d | Unknown14 |
| 42 | 4.512 | s | *β*-glucose | s | *β*-glucose |
| 43 | 4.627 | d | Cellobiose | d | Cellobiose |
| 44 | 4.683 | d | *α*-glucose | d | *α*-glucose |
| 45 | 5.651 | t | Unknown15 | x | Unknown15 |
| 46 | 5.808 | d | Uracil | d | Uracil |
| 47 | 5.885 | d | Unknown16 | d | Unknown16 |
| 48 | 5.918 | s | Unknown17 | s | Unknown17 |
| 49 | 5.933 | m | Unknown18 | m | Unknown18 |
| 50 | 6.307 | d | Unknown19 | d | Unknown19 |
| 51 | 6.322 | d | Unknown20 | d | Unknown20 |
| 52 | 6.542 | s | Fumarate | s | Fumarate |
| 53 | 6.920 | d | Tyrosine | d | Tyrosine |
| 54 | 7.026 | d | Unknown21 | d | Unknown21 |

**Table S2** 1D and 2D *J*-resolved ^1^H NMR Spectra of metabolic profiles of dill leaf extracts (continued)

| **No.** | **ppm** | **Multiplicity**  **by 1D NMR** | **1^st^ tentative metabolites** | **Multiplicity by**  **2D *J*-resolved ^1^H NMR** | **Final tentative metabolites** |
| --- | --- | --- | --- | --- | --- |
| 55 | 7.161 | s | Unknown22 | s | Unknown22 |
| 56 | 7.211 | d | Unknown23 | s | Unknown23 |
| 57 | 7.316 | m | Phenylalanine | m | Phenylalanine |
| 58 | 7.332 | bs | Tryptophan | bs | Tryptophan |
| 59 | 7.559 | s | Pyridoxal | s | Pyridoxal |
| 60 | 7.74 | d | Indole-3-lactate | d | Indole-3-lactate |
| 61 | 7.827 | d | Unknown24 | d | Unknown24 |
| 62 | 7.864 | d | Unknown25 | d | Unknown25 |
| 63 | 7.874 | d | Unknown26 | d | Unknown26 |
| 64 | 8.025 | s | N-formyl-L-aspartate | s | Unknown27 |
| 65 | 8.06 | s | Adenine | s | Adenine |
| 66 | 8.18 | s | Inosine | s | Inosine |
| 67 | 8.249 | s | Formate | s | Formate |
| 68 | 8.486 | s | Picolinate | x | Unknown28 |
| 69 | 8.55 | s | Folate | s | Folate |
