# Supplementary material for: Metabolomic analysis of bioactive compounds in dill (Anethum graveolens L.) extracts
Source: PeerJ. 2025 Jun 10;13:e19567. doi: 10.7717/peerj.19567 (PMC12164813; doi:10.7717/peerj.19567)
Supplement: Supplemental Information 4 [file peerj-13-19567-s004.docx]

**Table S3** Quantification of absolute concentration of dill-identified metabolites at different conditions

| **Metabolites** | **Metabolites concentration (mM)** | | | | | |
| --- | --- | --- | --- | --- | --- | --- |
|  | **Hot water (90 °C)** | | | **Room temperature water (27 °C)** | | |
|  | **2 min** | **1 h** | **2 h** | **2 min** | **1 h** | **2 h** |
| Pantothenate | 1.25 ± 0.39 | 0.94 ± 0.21 | 1.02 ± 0.24 | 1.20 ± 0.52 | 1.38 ± 0.67 | 1.54 ± 0.48 |
| Leucine | 1.27 ± 0.41 | 0.98 ± 0.21 | 1.04 ± 0.23 | 1.22 ± 0.53 | 1.47 ± 0.73 | 1.73 ± 0.52 |
| Isoleucine | 3.75 ± 1.21 | 2.71 ± 0.55 | 2.95 ± 0.67 | 3.26 ± 1.44 | 3.50 ± 1.76 | 4.01 ± 1.21 |
| Valine | 3.52 ± 1.13 | 2.56 ± 0.53 | 2.79 ± 0.63 | 3.00 ± 1.34 | 3.25 ± 1.62 | 3.72 ± 1.14 |
| *α*-ketoisovalerate | 0.21 ± 0.07 | 0.17 ± 0.04 | 0.17 ± 0.04 | 0.20 ± 0.08 | 0.21 ± 0.10 | 0.23 ± 0.07 |
| *β*-hydroxybutyrate | 0.22 ± 0.07 | 0.17 ± 0.03 | 0.19 ± 0.04 | 0.25 ± 0.13 | 0.22 ± 0.11 | 0.25 ± 0.07 |
| (*S*)-3-hydroxybutyrate | 2.45 ± 0.79 | 1.75 ± 0.35 | 1.90 ± 0.43 | 2.10 ± 0.94 | 2.24 ± 1.12 | 2.53 ± 0.76 |
| Threonine | 0.57 ± 0.19 | 0.41 ± 0.09 | 0.45 ± 0.10 | 0.50 ± 0.22 | 0.54 ± 0.26 | 0.61 ± 0.18 |
| Lactate | 3.06 ± 0.98 | 2.17 ± 0.46 | 2.31 ± 0.51 | 2.54 ± 1.18 | 2.76 ± 1.28 | 3.12 ± 0.87 |
| Alanine | 7.32 ± 2.36 | 5.36 ± 1.05 | 5.92 ± 1.37 | 6.22 ± 2.77 | 6.60 ± 3.17 | 7.54 ± 2.24 |
| Acetate | 4.74 ± 1.22 | 3.34 ± 0.71 | 3.95 ± 1.16 | 4.25 ± 1.93 | 4.39 ± 2.36 | 5.43 ± 1.79 |
| *α*-hydroxyisovalerate | 6.04 ± 1.90 | 4.61 ± 0.98 | 4.76 ± 1.03 | 5.61 ± 2.43 | 6.46 ± 3.12 | 7.10 ± 2.16 |
| Isovalerate | 3.76 ± 1.18 | 2.80 ± 0.60 | 2.94 ± 0.64 | 3.40 ± 1.48 | 3.93 ± 1.94 | 4.35 ± 1.34 |
| *α*-ketoisocaproate | 6.57 ± 2.07 | 4.91 ± 1.06 | 5.18 ± 1.14 | 6.06 ± 2.63 | 7.01 ± 3.38 | 7.71 ± 2.38 |
| Homocysteine | 4.12 ± 1.30 | 3.03 ± 0.62 | 3.21 ± 0.73 | 3.45 ± 1.52 | 3.75 ± 1.81 | 4.10 ± 1.24 |
| Methionine | 1.16 ± 0.36 | 0.86 ± 0.18 | 0.91 ± 0.20 | 0.97 ± 0.43 | 1.13 ± 0.55 | 1.30 ± 0.40 |
| *γ*-aminobutyrate | 7.88 ± 2.46 | 5.77 ± 1.20 | 6.19 ± 1.38 | 6.89 ± 3.00 | 7.77 ± 3.81 | 8.81 ± 2.64 |

**Table S3** Quantification of absolute concentration of dill-identified metabolites at different conditions (continued)

| **Metabolites** | **Metabolites concentration (mM)** | | | | | |
| --- | --- | --- | --- | --- | --- | --- |
|  | **Hot water (90 °C)** | | | **Room temperature water (27 °C)** | | |
|  | **2 min** | **1 h** | **2 h** | **2 min** | **1 h** | **2 h** |
| Succinate | 29.61 ± 9.45 | 20.44 ± 3.84 | 22.76 ± 5.32 | 23.97 ± 10.64 | 24.12 ± 12.20 | 28.86 ± 8.93 |
| Pyridoxamine | 5.66 ± 1.80 | 4.14 ± 0.80 | 4.36 ± 0.87 | 3.93 ± 1.86 | 4.75 ± 2.31 | 5.34 ± 0.68 |
| Citrate | 6.21 ± 1.96 | 4.68 ± 0.95 | 4.94 ± 1.05 | 3.72 ± 1.80 | 4.94 ± 2.31 | 5.44 ± 1.65 |
| Aspartate | 15.71 ± 4.85 | 11.34 ± 2.43 | 12.17 ± 2.51 | 11.16 ± 5.07 | 13.42 ± 6.43 | 14.83 ± 4.57 |
| Sarcosine | 0.50 ± 0.16 | 0.37 ± 0.08 | 0.38 ± 0.08 | 0.37 ± 0.16 | 0.43 ± 0.20 | 0.47 ± 0.14 |
| Acetylcholine | 16.01 ± 5.13 | 12.14 ± 2.56 | 13.01 ± 2.08 | 12.93 ± 5.84 | 15.96 ± 7.94 | 18.08 ± 5.58 |
| 3,7-dimethylurate | 2.41 ± 0.80 | 1.83 ± 0.46 | 1.89 ± 0.44 | 2.15 ± 0.92 | 2.40 ± 1.21 | 2.63 ± 0.80 |
| Proline | 1.54 ± 0.49 | 1.17 ± 0.26 | 1.26 ± 0.27 | 1.39 ± 0.61 | 1.49 ± 0.71 | 1.62 ± 0.49 |
| Sucrose | 54.39 ± 17.15 | 37.55 ± 7.88 | 39.30 ± 9.19 | 35.92 ± 16.48 | 32.67 ± 14.88 | 30.85 ± 8.49 |
| Malate | 15.07 ± 4.71 | 11.15 ± 2.29 | 12.02 ± 2.63 | 9.88 ± 4.56 | 12.24 ± 5.92 | 13.73 ± 4.19 |
| *β*-glucose^a^ | 0.52 ± 0.17 | 0.42 ± 0.09 | 0.45 ± 0.10 | 0.53 ± 0.23 | 0.76 ± 0.38 | 1.03 ± 0.30 |
| Cellobiose^a^ | 5.56 ± 1.70 | 4.32 ± 0.77 | 4.48 ± 0.99 | 8.14 ± 3.55 | 12.17 ± 6.89 | 15.15 ± 4.95 |
| *α*-glucose^a^ | 4.51 ± 1.42 | 3.38 ± 0.73 | 3.63 ± 0.80 | 5.95 ± 2.42 | 8.59 ± 4.51 | 11.45 ± 3.39 |
| Uracil | 0.26 ± 0.09 | 0.21 ± 0.04 | 0.22 ± 0.05 | 0.18 ± 0.08 | 0.22 ± 0.11 | 0.25 ± 0.08 |
| Fumarate | 4.53 ± 1.50 | 3.05 ± 0.61 | 3.37 ± 0.94 | 4.02 ± 1.88 | 3.85 ± 2.06 | 4.73 ± 1.50 |
| Tyrosine | 1.00 ± 0.31 | 0.80 ± 0.17 | 0.85 ± 0.17 | 0.63 ± 0.29 | 0.78 ± 0.41 | 0.91 ± 0.30 |
| Phenylalanine | 0.85 ± 0.27 | 0.66 ± 0.15 | 0.70 ± 0.14 | 0.75 ± 0.33 | 0.82 ± 0.40 | 0.91 ± 0.28 |

**Table S3** Quantification of absolute concentration of dill-identified metabolites at different conditions (continued)

| **Metabolites** | **Metabolites concentration (mM)** | | | | | |
| --- | --- | --- | --- | --- | --- | --- |
|  | **Hot water (90 °C)** | | | **Room temperature water (27 °C)** | | |
|  | **2 min** | **1 h** | **2 h** | **2 min** | **1 h** | **2 h** |
| Tryptophan | 1.43 ± 0.46 | 1.12 ± 0.24 | 1.20 ± 0.25 | 1.18 ± 0.53 | 1.30 ± 0.65 | 1.48 ± 0.45 |
| Pyridoxal^b^ | 0.90 ± 0.28 | 0.78 ± 0.16 | 0.84 ± 0.18 | 0.27 ± 0.14 | 0.27 ± 0.12 | 0.28 ± 0.10 |
| Indole-3-lactate^b^ | 0.17 ± 0.06 | 0.16 ± 0.04 | 0.16 ± 0.05 | 0.06 ± 0.03 | 0.11 ± 0.05 | 0.12 ± 0.04 |
| Adenine^b^ | 0.11 ± 0.04 | 0.16 ± 0.04 | 0.17 ± 0.03 | 0.03 ± 0.02 | 0.03 ± 0.01 | 0.04 ± 0.02 |
| Inosine^b^ | 0.15 ± 0.05 | 0.16 ± 0.04 | 0.18 ± 0.03 | 0.03 ± 0.01 | 0.05 ± 0.03 | 0.06 ± 0.02 |
| Formate | 5.95 ± 1.84 | 4.16 ± 0.82 | 4.59 ± 1.00 | 4.98 ± 2.32 | 5.01 ± 2.53 | 6.03 ± 1.91 |
| Folate^b^ | 0.13 ± 0.05 | 0.16 ± 0.05 | 0.17 ± 0.03 | 0.01 ± 0.01 | 0.01 ± 0.01 | 0.02 ± 0.01 |

^a^ Metabolites presented in hot water (90 °C) than RT water (27 °C) conditions, ^b^ Metabolites presented in RT water than hot water conditions, data are represented in means ± SD (n = 5).
